# Phytocannabinoid Compositions from Cannabis Act Synergistically with PARP1 Inhibitor against Ovarian Cancer Cells In Vitro and Affect the Wnt Signaling Pathway

**DOI:** 10.3390/molecules27217523

**Published:** 2022-11-03

**Authors:** Nurit Shalev, Michelle Kendall, Seegehalli M. Anil, Sudeep Tiwari, Hadar Peeri, Navin Kumar, Eduard Belausov, Ajjampura C. Vinayaka, Hinanit Koltai

**Affiliations:** 1 The Mina and Everard Goodman, Faculty of Life Sciences, Bar-Ilan University, Ramat Gan 5290002, Israel; 2 Institute of Plant Science, Agricultural Research Organization, Volcani Center, Rishon LeZion 7505101, Israel; 3 Canna Onc Research, Santa Barbara, CA 93101, USA

**Keywords:** ovarian cancer, cannabis, phytocannabinoids, apoptosis, cytotoxicity, Wnt pathway, PARP1

## Abstract

Ovarian cancer (OC) is the single most lethal gynecologic malignancy. *Cannabis sativa* is used to treat various medical conditions, and is cytotoxic to a variety of cancer types. We sought to examine the effectiveness of different combinations of cannabis compounds against OC. Cytotoxic activity was determined by XTT assay on HTB75 and HTB161 cell lines. Apoptosis was determined by flow cytometry. Gene expression was determined by quantitative PCR and protein localization by confocal microscopy. The two most active fractions, F5 and F7, from a high Δ9–tetrahydrocannabinol (THC) cannabis strain extract, and their standard mix (SM), showed cytotoxic activity against OC cells and induced cell apoptosis. The most effective phytocannabinoid combination was THC+cannabichromene (CBC)+cannabigerol (CBG). These fractions acted in synergy with niraparib, a PARP inhibitor, and were ~50-fold more cytotoxic to OC cells than to normal keratinocytes. The F7 and/or niraparib treatments altered Wnt pathway-related gene expression, epithelial–mesenchymal transition (EMT) phenotype and β-catenin cellular localization. The niraparib+F7 treatment was also effective on an OC patient’s cells. Given the fact that combinations of cannabis compounds and niraparib act in synergy and alter the Wnt signaling pathway, these phytocannabinoids should be examined as effective OC treatments in further pre-clinical studies and clinical trials.

## 1. Introduction

Ovarian cancer (OC) is the second most common and the most lethal gynecologic malignancy in the western world; about 70% of cases are diagnosed at an advanced stage. Late-stage ovarian cancer is incurable in the majority of cases [1]. Worldwide, there were over 300,000 cases of OC (1.6% of all cancers) and 200,000 deaths (2.1% of all cancers) in 2020 [2]. 

Epithelial OC typically emerges in postmenopausal women, with a few months of abdominal pain and distension, vague and subtle symptoms that are often disregarded. Many women go six months before being properly diagnosed. There is no screening test for OC and population level monitoring does not reduce mortality [3]. Most women have advanced disease, for which the standard of care remains surgery and platinum-based cytotoxic chemotherapy [4]. In about 80% of the cases, disease relapse is expected, on average after 24 months, and, ultimately, multi-drug resistance develops, with very few women surviving five years after diagnosis.

*Cannabis sativa* is being used worldwide to alleviate numerous symptoms associated with various medical conditions [5]. Each *C. sativa* strain produces several dozen compounds, and, in total, the species produces around 600 different molecules, including more than 150 phytocannabinoids and hundreds of terpenes [6,7,8]. Recently, a large number of studies have demonstrated that many phytocannabinoids possess in vitro and in vivo anticancer activity, and inhibit, e.g., cell proliferation, migration, and angiogenesis, as well as induce apoptosis in prostate, skin, lung, glioma and breast cancer cells [9,10,11].

However, only a few studies have examined the effectiveness of cannabis compounds against OC. Cannabidiol (CBD) was shown to have anti-proliferative activity in an OC cell line and in a chick embryo model (i.e., in ovo), and administration of CBD in solution, or carried by nanoparticles in combination with paclitaxel, increased paclitaxel effectivity both in vitro and in ovo [12,13]. In a single patient case study, the use of Laetrile and ‘CBD oil’ improved the expression of markers associated with low-grade serous ovarian cancer [14]. However, ‘CBD oil’ may contain multiple compounds extracted from cannabis in addition to CBD, and the actual combination(s) of cannabis molecules that appear effective against OC were not identified. 

Aberrant activation of the canonical wingless/int1 (Wnt) pathway was suggested to have a critical role in OC development [15]. Wnt signaling maintains compartments of diverse adult stem cells and is associated with chemotherapy resistance in cancer [16]. The Wnt signaling pathway plays a role in epithelial–mesenchymal transition (EMT). During EMT transition epithelial cells lose polarity, cell–cell contact, and other morphological characteristics of epithelial cells and acquire the features of mesenchymal cells, including increased motility [17]. The Wnt signaling pathway is activated by the interaction of Wnt with Frizzled (FZD) receptors, resulting in the eventual accumulation of β-catenin in the cytosol and its migrations to the nucleus, where β-catenin regulates target gene expression [17]. Dickkopf-related protein (DKK) acts as an inhibitor of Wnt signaling [17]. In OC, for example, FZD1 expression was increased in epithelial ovarian cancer [18]. 

Here, we identify fractions of cannabis extract and combinations of cannabis compounds that are cytotoxic to OC cells. These combinations of cannabis compounds and fractions lead to cell apoptosis. Moreover, these fractions act in synergy with niraparib in vitro and the synergetic mixture of niraparib and cannabis fractions are ~50-fold more cytotoxic to OC cells than to normal keratinocytes, and alter components of the Wnt signaling pathway.

## 2. Results

### 2.1. Determining Cannabis Strains with Cytotoxic Activity against Ovarian Cancer Cell Lines

**Figure 1:**
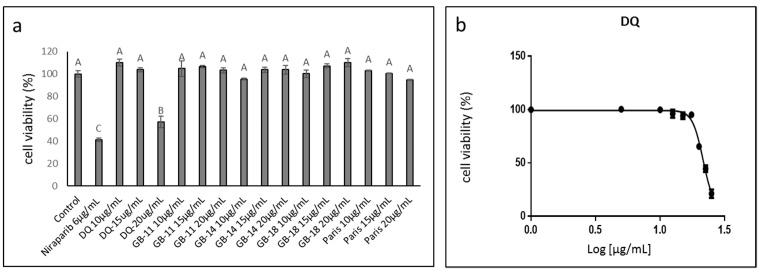
(**a**) Cell viability of HTB75 cells following treatment with the crude extract of cannabis strains DQ, GB-11, GB-14, GB-18 and Paris. Cell viability was determined by XTT assay as a function of live cell number at 48 h. Control was the vehicle-treated control (1.00% *v*/*v* methanol). Error bars indicate ± SE (*n* = 3). Levels with different Upper case letters were significantly different from all combinations of pairs according to the Tukey–Kramer honest significant difference (HSD; *p* ≤ 0.05). (**b**) Cell viability following treatment with *C. sativa* DQ crude extract at different concentrations for IC50 value calculation from 5P logistic curve fit using GraphPad Prism version 6.1. Concentration 0 was the vehicle-treated control (1.25% *v*/*v* methanol). Error bars indicate ± SE (*n* = 3).

Extracts of several *C. sativa* strains were examined for cytotoxic activity against ovarian cancer cell line HTB75. DQ (IMC, Glil-Yam, Israel), a high Δ9–tetrahydrocannabinol (THC) strain, was the most effective at the highest examined concentration (20 µg/mL) resulting in ~50% cell death (Figure 1a). Other strains examined included GB-11 (THC:CBD: cannabigerol [CBG] 22:40:30, Israel Gene Bank [IGB]), GB-14 (THC:CBD:CBG 55:9:28, IGB), and GB-18 (High THC, IGB) and Paris (THC:CBD 22:35, IMC). No strains other than DQ showed cytotoxic activity against the OC cells, even at 20 µg/mL (Figure 1a). The IC50 for the DQ crude extract was determined to be 21.51 µg/mL (Figure 1b).

### 2.2. Identifying the Active Fractions of the DQ Cannabis Strain

**Figure 2:**
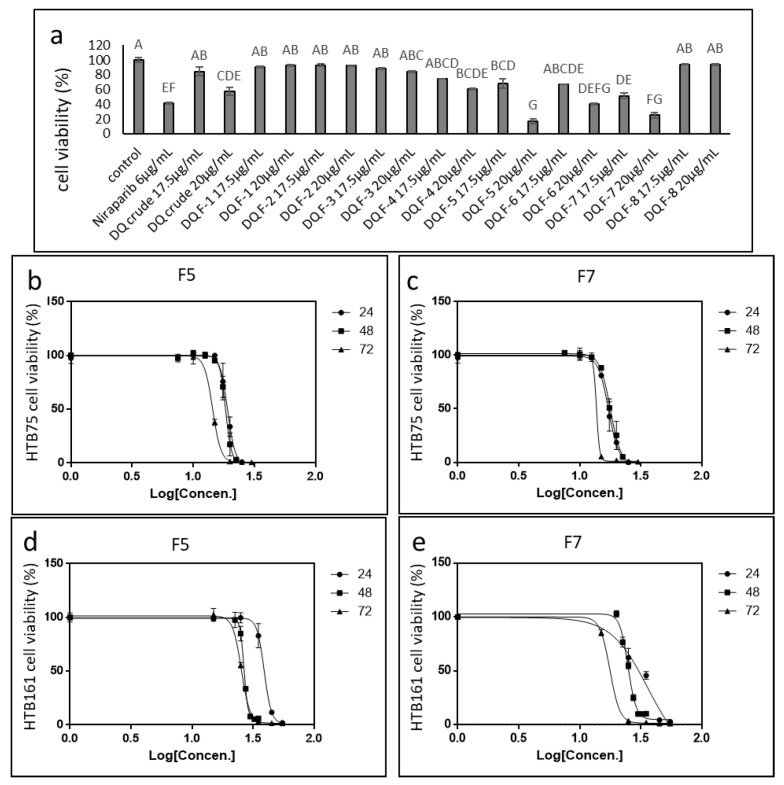
(**a**) Cell viability of HTB75 cells following treatment with DQ extract fractions F1–F8, and the crude extract. Cell viability was determined by XTT assay as a function of live cell number. Control is the vehicle-treated control (1.00% *v*/*v* methanol). Error bars indicate ± SE (*n* = 3). Levels with different Upper case letters were significantly different from all combinations of pairs according to the Tukey–Kramer honest significant difference test (HSD; *p* ≤ 0.05). (**b**,**c**) Cell viability of HTB75 cells following treatment with *C. sativa* DQ fractions F5 and F7 (respectively) at different concentrations for 24 h, 48 h and 72 h. (**d**,**e**) Cell viability of HTB161 cells following treatment with *C. sativa* DQ fractions F5 and F7 (respectively) at different concentrations for 24 h, 48 h and 72 h. IC50 values calculation was done from logistic curve fit using GraphPad Prism version 6.1. Concentration 0 was the vehicle-treated control (1.50% *v*/*v* methanol). Error bars indicate ± SE (*n* = 3).

The most active extract, DQ, was fractionated as described in [19]. The activity of the different fractions was tested on HTB75 cells. Four of the 8 fractions (F4, F5, F6 and F7) showed significant cytotoxic activity at the examined concentrations. F5 and F7 were the most active, with the highest examined concentration (20 µg/mL) resulting in ~80% cell death (Figure 2a). F6 and F4 were less active, with the highest examined concentration (20 µg/mL) resulting in ~60% and ~40% cell death, respectively (Figure 2a). Treatments with F1–F3 and F8 showed no cytotoxic activity against HTB75 cells (Figure 2a). 

The IC50 values of the most active fractions in HTB75 were determined to be 18.90 and 17.17 µg/mL for F5 and F7 at 24 h, respectively, 18.36 and 16.95 µg/mL for F5 and F7 at 48 h, respectively, and 14.31 and 13.07 µg/mL for F5 and F7 at 72 h, respectively (Figure 2b,c). 

Cytotoxicity for F5 and F7 was examined in another OC cell line, HTB161. The IC50 values for F5 and F7 were 37.87 and 30.46 µg/mL for F5 and F7 at 24 h, respectively, 26.26 and 24.46 µg/mL for F5 and F7 at 48 h, respectively, and 25.34 and 17.66 µg/mL for F5 and F7 at 72 h, respectively (Figure 2d,e). In HTB76, IC50 values at 48 h were 29.12 and 23.79 µg/mL for F5 and F7, respectively (Supplementary Figure S1). 

The F5 and F7 phytocannabinoid composition is described in [19]. F5 contained mainly THC and cannabinol (CBN) and CBG to a lesser extent, while F7 included mainly THC and cannabichromene (CBC), with smaller proportions of CBN and CBG, as well as traces of other phytocannabinoids [19]. The terpene composition of F5 is reported in [19]; the terpene composition of F7 analyzed by GC/MS is reported in Supplementary Table S1. 

### 2.3. Determining the Activity of the Phytocannabinoid F5 and F7 Standard Mixes

**Figure 3:**
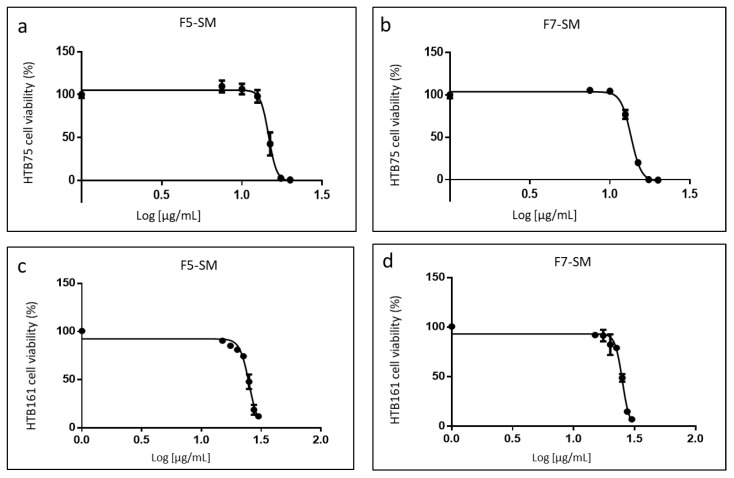
Cell viability of HTB75 (**a**,**b**) and HTB161 (**c**,**d**) cells following treatment with F5-SM and F7-SM at different concentrations for IC50 values at 48 h calculation from 5P logistic curve fit using GraphPad Prism version 6.1. Concentration 0 was the vehicle-treated control (2.50% and 3.50% *v*/*v* methanol for HTB75 and HTB161 respectively). Error bars indicate ± SE (*n* = 3).

In order to confirm the active compositions of F5 and F7, standard mixes (SM) of phytocannabinoid standards were formulated as closely as possible to the original fraction compositions. On HTB75 cells, F5-SM had an IC50 of 14.67 µg/mL (Figure 3a), which was lower than that of F5 (18.36 µg/mL; Figure 2b). F7-SM showed an IC50 value of 13.56 µg/mL (Figure 3b), again lower than that of F7 (16.95 µg/mL; Figure 2c). On HTB161 cells F5-SM had an IC50 of 25.11 µg/mL (Figure 3c), which was lower than that of F5 (26.26 µg/mL; Figure 2d). F7-SM showed an IC50 value of 25.09 µg/mL (Figure 3d), higher than that of F7 (24.46 µg/mL; Figure 2e). IC50 values for HTB76 were 20.38 and 20.66 µg/mL for F5-SM and F7-SM, respectively (Supplementary Figure S1). All IC50 values are summarized in Supplementary Table S2. 

### 2.4. Determining the Most Effective Combinations of Phytocannabinoids

**Figure 4:**
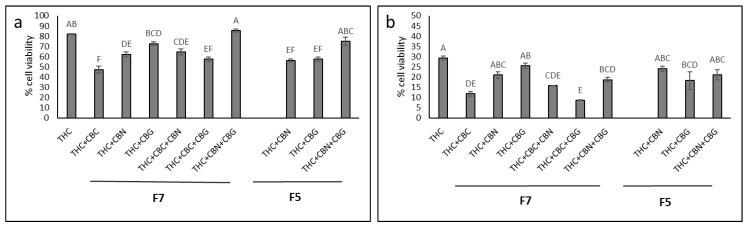
Cell viability of HTB75 cells following treatment with THC or with combinations of THC, CBG, CBC and CBN as in F7 or F5, at total concentrations of (**a**) 13 and (**b**) 15 µg/mL (treatments are listed in Supplementary Table S3). Cell viability was determined by XTT assay as a function of live cell number at 48 h. All calculations were in relation to the control (the vehicle treated control of 1.30% and 1.50% *v*/*v* methanol for a and b, respectively) that was considered to have 100% cell viability (not shown). Error bars indicate ± SE (*n* = 3). Levels with different Upper case letters were significantly different from all combinations of pairs by Tukey–Kramer honest significant difference (HSD; *p* ≤ 0.05).

To determine the most effective combinations of the main phytocannabinoids [8] in F5 and F7, combinations of THC, CBG, CBC and CBN standards were examined on HTB75 cells at ratios found in F5 or F7, at a fixed total concentration, in comparison to THC (Figure 4). At concentrations of 13 and 15 µg/mL (Figure 4a,b, respectively), THC alone was less effective than some of the various combinations of THC, CBC, CBG and CBN at ratios found in F5 or F7 (Figure 4). The most effective combinations at both 13 and 15 µg/mL were those of THC+CBC and THC+CBC+CBG at ratios found in F7 (ratios of 7.5:2.5 and 7.4:2.5:0.1, respectively; Figure 4). For the F5 combination-ratio, THC+CBG was the most effective treatment, but was not significantly different from the other F5-based combinations (Figure 4).

### 2.5. Determining the Effects of Treatment on Cell Apoptosis

**Figure 5:**
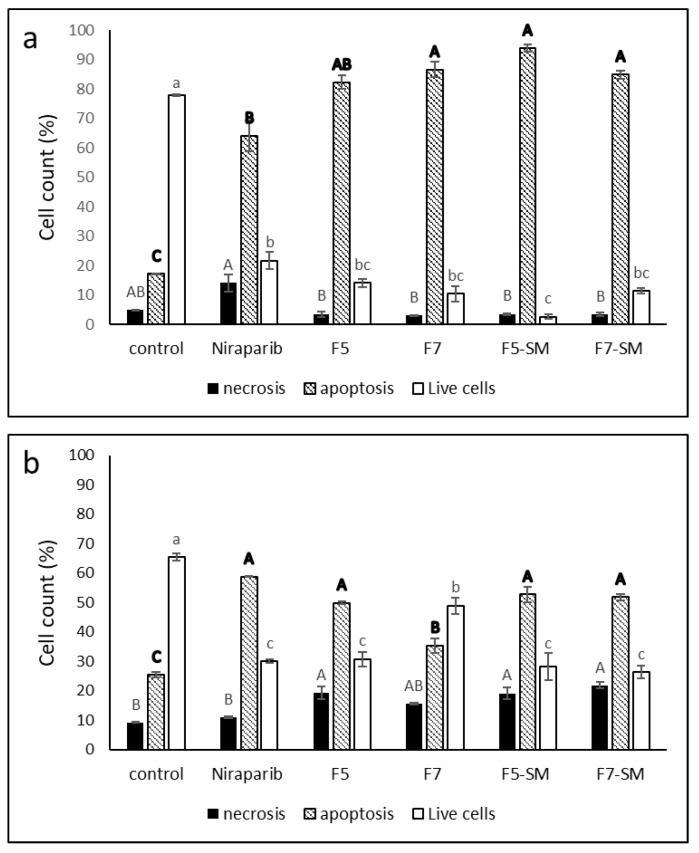
Percentage of viable, apoptotic, or necrotic, HTB75 cells (**a**) or HTB161 cells (**b**) following treatment with niraparib (5.1 µg/mL and 24.8 µg/mL respectively), F5 (19.1 µg/mL and 26.2 µg/mL respectively), F7 (19.4 µg/mL and 24.5 µg/mL respectively), F5-SM (17.4 µg/mL and 30.1 respectively), or F7-SM (16.3 µg/mL and 30.1 µg/mL respectively) for 48 h. 10^4^ cells were analyzed per treatment. Control was vehicle control (1% methanol or 0.5% DMSO *v*/*v* for HTB75 and 3% methanol or 1.2% DMSO *v*/*v* for HTB161). The treated cells were harvested and analyzed by flow cytometry following annexin V-FITC/PI staining. Error bars indicate ± SE (*n* = 3). Levels with different Upper or lower case letters of similar font and style were significantly different from all combinations of pairs according to the Tukey–Kramer honest significant difference (HSD; *p* ≤ 0.05).

Treatment of HTB75 cells with F5 and F7 for 48 h led to 83.3% and 88.0% cell apoptosis, respectively, in comparison to 17.3% apoptosis in the vehicle control. Treatment with the phytocannabinoid standard mixes, F5-SM and F7-SM, led to similarly high levels of cell apoptosis: 93.9% and 85.0%, respectively (Figure 5a). The chemotherapy drug niraparib led to 64.1% apoptotic cells (Figure 5a). Only low levels of necrosis were recorded with the cannabis treatments, similar to the control (Figure 5a). The non-treated control demonstrated apoptosis values similar to the vehicle control (e.g., 78.6 and 78.0% live cells, 17.6 and 17.1% apoptosis and 3.8 and 4.9% necrosis in the non-treated and vehicle control, respectively). Similar results, suggesting significant induction of apoptosis, were evident for HTB161 treatments (Figure 5b). However, in this cell line the niraparib only treatment was the most effective in induction of apoptosis and there was an increased proportion of live cells in comparison to HTB75 and some increase in cell necrosis, mainly with F5, F5-SM and F7-SM treatments (Figure 5b). 

### 2.6. Determining the Synergistic Effects of CB1 and CB2 Receptor Inverse Agonists, a TRPA1 Receptor Blocker, and TRPV1 or TRPV2 Receptor Antagonists on Cytotoxic Activity

**Figure 6:**
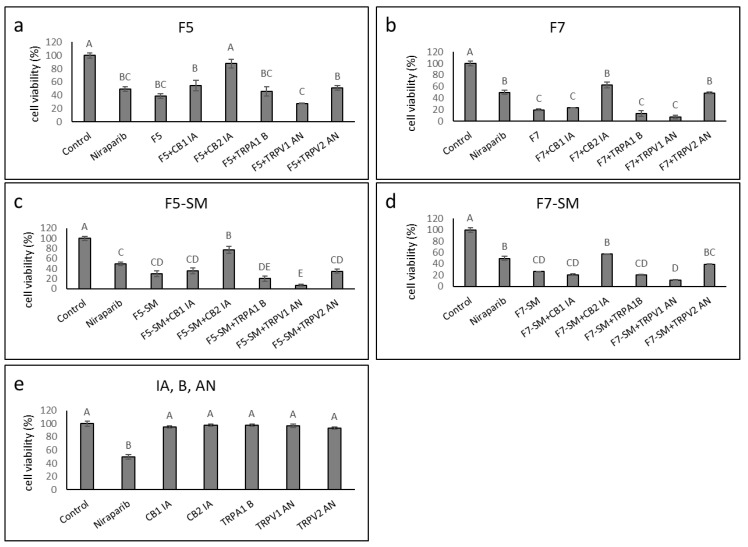
Cell viability of HTB75 cells following treatment with F5 (**a**), F7 (**b**), F5-SM (**c**) or F7-SM (**d**), with or without CB1 and CB2 inverse agonists (IA), a TRPA1 blocker (B), and TRPV1 or TRPV2 antagonists (AN) for 48 h. Cells were treated with F5 (19.1 µg/mL), F7 (19.4 µg/mL), F5-SM (14.7 µg/mL) or F7-SM (13.6 µg/mL), with or without the receptor IA, B or AN (10 µM). (**e**) The effects of IA, B or AN on viability of cells. Cell viability was determined by XTT assay at 48 h as a function of live cell number. Niraparib (6 µg/mL) served as a positive control. Control was vehicle control (1.47% *v*/*v* methanol + 1.00% *v*/*v* DMSO). Error bars indicate ± SE (*n* = 3). Levels with different Upper case letters were significantly different from all combinations of pairs according to the Tukey–Kramer honest significant difference (HSD; *p* ≤ 0.05).

We determined the effect of adding CB1 or CB2 inverse agonists (IA), TRPV1 or TRPV2 antagonists (AN) or a TRPA1 blocker (B) on F5, F7, F5-SM and F7-SM activity. HTB75 cells were treated with F5, F7, F5-SM or F7-SM with or without IA, AN or B. In the presence of CB2 IA, the cytotoxic effect of F5, F7, F5-SM or F7-SM was significantly reduced (87.7, 62.7, 77.1, 57.1%, viable cells respectively, vs. 38.5, 19.3, 29.6, 26.3% viable cells without CB2 IA); Figure 6a–d). Furthermore, TRPV2 AN co-treatment with F7 and F7-SM interfered, to some extent, with their activities (significantly with F7; Figure 6b,d). Cell viability percentages for treatments with F7 or F7-SM were 48.9 or 39.2% with TRPV2 AN vs. 19.3 or 26.3% without, respectively (Figure 6b,d). Treatments with F5, F5-SM, F7 or F7-SM in the presence of TRPV1 AN, TRPA1 B or CB1 IA, did not significantly reduce the cytotoxicity of the treatments (Figure 6a–d). Treatment with TRPV1 AN even increased the cytotoxicity of F5-SM (Figure 6c). When applied without cannabis fractions or compounds, none of the inverse agonists, blockers, or agonists affected HTB75 cell viability (Figure 6e). Similar results for the CB2 receptor were obtained also for the HTB161 cells: CB2 IA substantially reduced F7 activity (Supplementary Figure S2). However, unlike the results for HTB75, CB1 IA, TRPA1 B, TRPV1 AN, and TRPV2 AN significantly increased the activity of F7 (Supplementary Figure S2a). When applied alone to HTB161 cells, CB2 IA reduced cell viability, to some extent, whereas TRPV1 AN increased it (Supplementary Figure S2b). 

The expression of the CB2 and TRPV2 receptors in HTB75 cells was not significantly changed by treatment with F5 or F7 (Table 1).

### 2.7. Determining the Effects of Combined Treatments of Cannabis Fractions or Compounds with Chemotherapy Drugs

**Figure 7:**
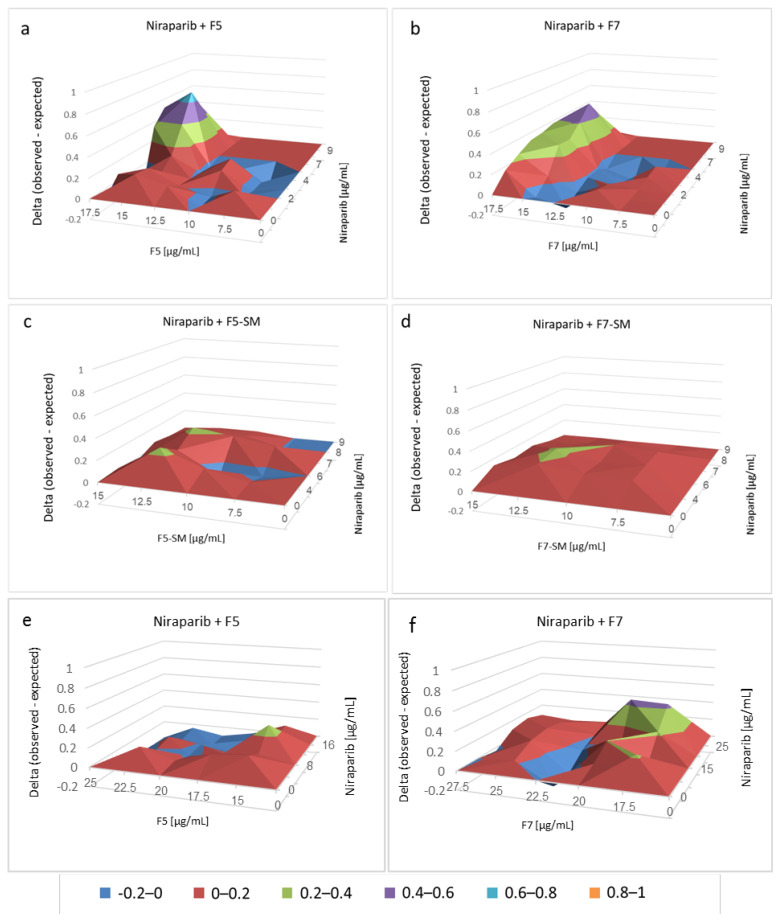
Synergistic interactions between F5, F7, F5-SM or F7-SM with niraparib on HTB75 cell viability at 48 h (**a**–**d**) and F5 or F7 with niraparib on HTB161 cell viability at 48 h (**e**,**f**) following combined treatments. Synergy of cytotoxic activity was calculated, based on the Bliss independence drug interaction model. Control was vehicle control (1.50% *v*/*v* methanol + 0.90% *v*/*v* DMSO for HTB75 and 1.38% *v*/*v* methanol + 2.50% *v*/*v* DMSO for HTB161). Synergy was apparent when the experimental (observed) value of cell survival inhibition was higher than the calculated (expected) value. Delta values of observed minus expected are shown on the *Y* axis. Supplementary Tables S4 and S5 present the significantly different synergistic delta values of F5, F7, F5-SM or F7-SM with niraparib from all pair combinations according to the Tukey–Kramer honest significant difference test (*n* = 3; HSD; *p* ≤ 0.05).

Chemotherapy drugs were examined for combined activity with F5, F7, F5-SM and F7-SM. Considerable and significant synergy (on a scale of 0 [no synergy] to 1 [high synergy]) was obtained for combinations of niraparib with F5 on HTB75 cells (with peaks of 0.6–0.8 and significant synergy at 15 or 17.5 µg/mL F5 and 6–8 µg/mL niraparib; Figure 7a; Supplementary Table S4). Considerable and significant synergy was obtained also with combinations of niraparib and F7 (with peaks of 0.4–0.6, respectively, and significant synergy of 15 or 17.5 µg/mL F7 and 3–8 µg/mL niraparib; Figure 7a; Supplementary Table S4). Synergy with F5-SM or F7-SM, on the other hand, was restricted to 0.2–0.4 only (Figure 7a; Supplementary Table S4). Synergistic interaction between F7 and niraparib was evident on the HTB161 cell line as well, with peaks of 0.4–0.6; significant synergy was evident at 17.5 or 20 µg/mL F7 and 25 µg/mL niraparib (Figure 7f; Supplementary Table S5). With F5, however, only low synergy was evident in co-treatments with niraparib (Figure 7e; Supplementary Table S5).

Gemcitabine showed low levels of synergy with the plant fractions (peaks of 0–0.2), and, in some cases, inhibited activity in the HTB75 cells (e.g., for 12.5 µg/mL F5 or F7 and 5–100 ng/mL gemcitabine; Supplementary Figure S3; Supplementary Table S6). Synergy was evident only with treatment of gemcitabine and F5-SM (a single peak of 0.2–0.4 at 10 µg/mL of F5-SM and 2.5 ng/mL of gemcitabine; Supplementary Figure S3 and Table S6).

### 2.8. Determining the Effects of Treatment on Normal Cells

**Figure 8:**
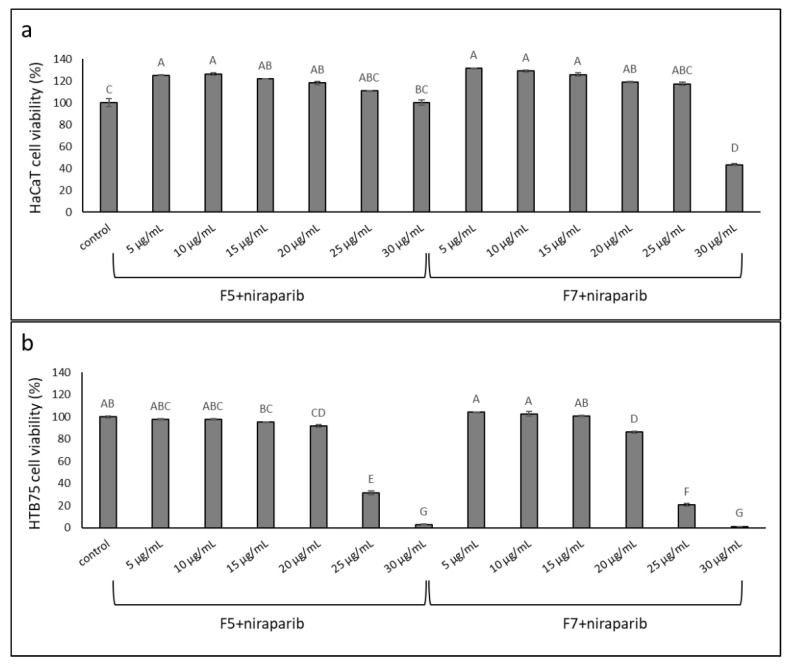
Cell viability of (**a**) HaCaT and (**b**) HTB75 cells following treatment at full confluence with niraparib:F5 or niraparib:F7 at ratio of 3.5:6.5, respectively, at different concentrations. Cell viability was determined by XTT assay at 48 h as a function of live cell number. Control was vehicle control (1% *v*/*v* methanol + 1% *v*/*v* DMSO). Error bars indicate ± SE (*n* = 3). Levels with different Upper case letters were significantly different from all combinations of pairs by Tukey–Kramer honest significant difference (HSD; *p* ≤ 0.05).

The cytotoxic activity of the synergistic mixture of niraparib+F5 and niraparib+F7 was assessed on normal cells using the keratinocyte (HaCaT) cell line. The synergistic ratio of mixture of niraparib:fraction (~3.5:6.5, derived from 8 µg/mL niraparib + 15 µg/mL F5 or F7; Figure 7a) was examined at several different concentrations; activity was examined in parallel on HaCaT and HTB75 cells at complete confluence (Figure 8). Some minor cell proliferation was apparent in HaCaT, but not in HTB75 cells, under treatments with low concentrations (Figure 8). At cytotoxic concentrations of the niraparib+F5 or niraparib+F7 mixtures (i.e., 8.7 µg/mL niraparib + 16.3 µg/mL F5 or F7 and 10.4 µg/mL niraparib + 19.6 µg/mL F5 or F7) considerably higher cytotoxicity was apparent against the cancer cells than against normal cells. In the most effective combined treatments of niraparib+F5 or niraparib+F7 (10.4 µg/mL niraparib + 19.6 µg/mL F5 or F7) this activity was ~50-fold higher on HTB75 than on HaCaT cells (Figure 8). 

### 2.9. Determining the Effects of Treatment on Wnt Pathway-Related Gene Expression

**Figure 9:**
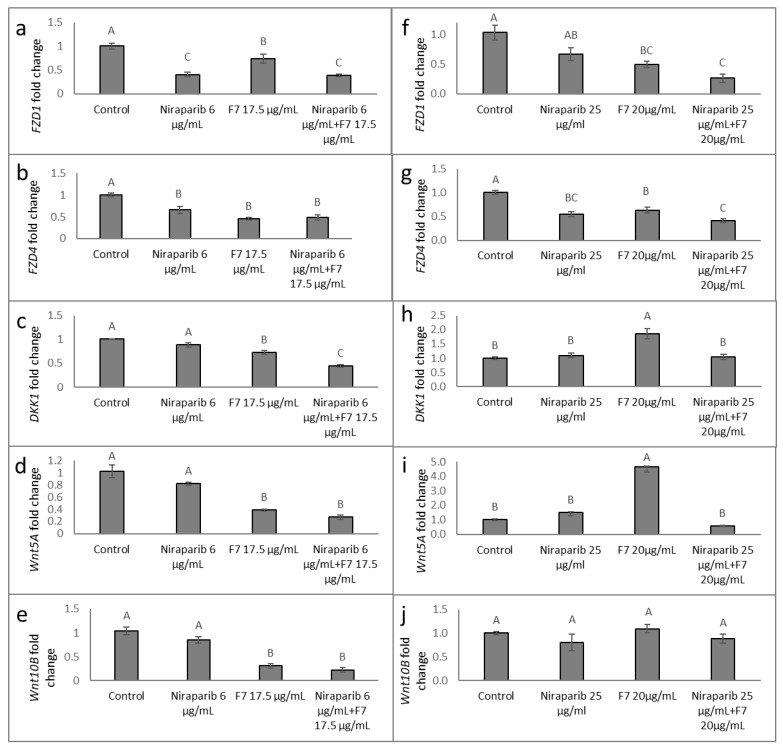
Quantitative PCR-based determination of the RNA steady state level in HTB75 (**a**–**e**) and HTB161 (**f**–**j**) cell lines of members of the *FZD*, *DKK* or *Wnt* gene families at 6 h of treatment with niraparib, F7, or a combination of niraparib+F7 relative to control. Gene transcript values were determined by quantitative PCR as a difference between the target gene versus a reference gene (HPRT). Values were calculated relative to the average expression of target genes in treated versus control using the 2^−ΔΔCt^ method. Control was the vehicle control (1.00% *v*/*v* methanol + 0.60% *v*/*v* DMSO). Error bars indicate ± S.E. (*n* = 3). Levels with different Upper case letters with the same font and style were significantly different from all combinations of pairs by Tukey-Kramer honest significant difference (HSD; *p* ≤ 0.05).

Gene expression of members of the *FZD* receptors, *DKK* or *Wnt* gene families were examined at 6 h after treatment (as described in [20]). In HTB75, treatments with F7 or niraparib and niraparib+F7 reduced *FZD4* and *FZD1* expression (Figure 9a,b). Similarly, in HTB161, the expression of *FZD4* and *FZD1* was significantly downregulated by all examined treatments, and mainly by niraparib+F7 (Figure 9f,g). 

In HTB75, *DKK1* expression was significantly reduced by the F7 and the combined niraparib+F7 treatments (Figure 9c). *DKK1* expression was induced in HTB161 by the F7 treatment (Figure 9h). Expression of *Wnt5A* was reduced by the F7 and niraparib+F7 treatments in HTB75 (Figure 9d) but was increased by the F7 treatment in HTB161 (Figure 9i). Expression of *Wnt10B* was reduced in HTB75, especially by the F7 and combined treatment (Figure 9e), and was unchanged to control in HTB161 (Figure 9j).

*RAD51* expression was examined at 6 h and again at 9 h post-treatment, the latter time since its expression was downstream and dependent on Wnt/β-catenin signaling activity [16]. In HTB161 *RAD51* expression was significantly reduced at 6 and 9 h by all treatments (Table 2). However, its expression was not significantly altered in HTB75 at the examined time points (Table 2). 

**Figure 10:**
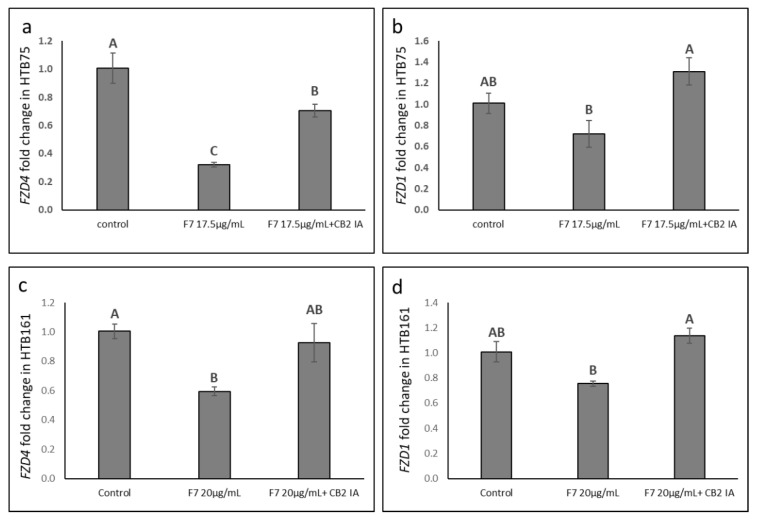
Quantitative PCR-based determination of the RNA steady state level in HTB75 (**a**,**b**) and HTB161 (**c**,**d**) cell lines of *FZD4* and *FZD1* genes at 6 h of treatment with F7 or F7+CB2 IA, relative to control. Gene transcript values were determined by quantitative PCR as a difference between the target gene versus a reference gene (HPRT). Values were calculated relative to the average expression of target genes in treated versus control using the 2^−ΔΔCt^ method. Control was the vehicle control (1.00% *v*/*v* methanol + 0.10% *v*/*v* DMSO). Error bars indicate ± S.E. (*n* = 3). Levels with different Upper case letters were significantly different from all combinations of pairs by Tukey-Kramer honest significant difference (HSD; *p* ≤ 0.05).

Co-treatment of HTB75 or HTB161 cells with F7 and CB2 IA diminished the repression F7 treatment had on *FZD* gene expression (Figure 10). 

### 2.10. Determining the Effects of Treatment on Epithelial-Mesenchymal Transition Phenotype

**Figure 11:**
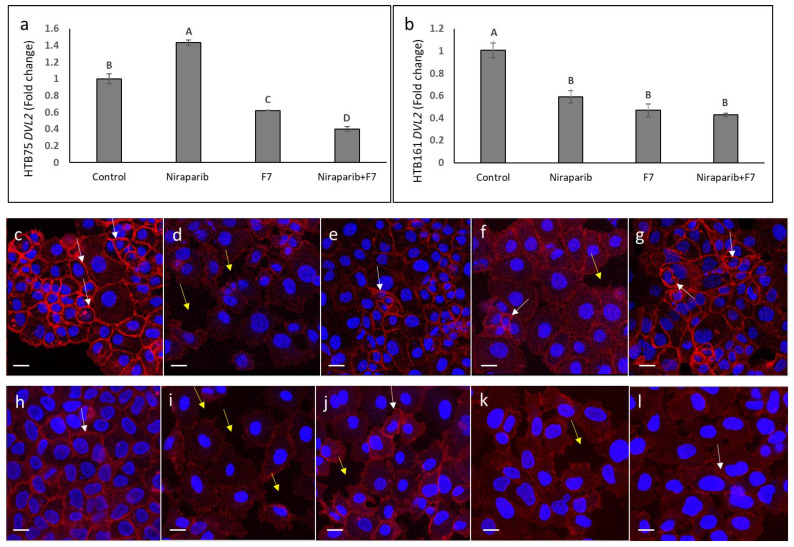
Quantitative PCR-based determination of the RNA steady state level in HTB75 (**a**) and HTB161 (**b**) cell lines of members of *DVL2* at 6 h of treatment with niraparib (at 6 or 25 µg/mL for HTB75 and HTB161, respectively), F7 (at 17.5 or 20 µg/mL for HTB75 and HTB161, respectively) or a combination of niraparib+F7 (6 + 17.5 and 25 + 20 µg/mL for HTB75 and HTB161, respectively), relative to control. Gene transcript values were determined by quantitative PCR as a difference between the target gene versus a reference gene (HPRT). Values were calculated relative to the average expression of target genes in treated versus control using the 2^−ΔΔCt^ method. Control was the vehicle control (1.00% *v*/*v* methanol + 0.60% *v*/*v* DMSO). Error bars indicate ± S.E. (*n* = 3). Levels with different Upper case letters with the same font and style were significantly different from all combinations of pairs by Tukey-Kramer honest significant difference (HSD; *p* ≤ 0.05). Representative examples of confocal images of HTB161 (**c**–**g**) and HTB75 (**h**–**l**) non-induced (**c**,**h**), induced cell treated with vehicle control (0.6% methanol + 0.3% DMSO and 0.7% methanol + 0.88% DMSO respectively; (**d**,**i**), induced cells following treatment with niraparib (3 and 17.5 µg/mL respectively; (**e**,**j**) or F7 (3 and 14 µg/mL, respectively; (**f**,**k**) and niraparib+F7 (3 + 3 and 17.5 + 14 µg/mL, respectively; (**g**,**l**)). β-catenin was detected using β-catenin monoclonal antibody PE, REAfinity™ (red color) and nuclei, stained using EasyProbes™ Hoechst (blue stain); *n* = 3, in each biological replicate multiple cells, were examined. Bars denote 20 µm. White arrows denote β-catenin signaling in cell membranes; yellow arrows denote spaces between cells in the monolayer.

We determined the effects of niraparib, F7 and niraparib+F7 treatments on the epithelial–mesenchymal transition (EMT) phenotype. Examination of EMT was based on *Dishevelled2* (*DVL2*) gene expression and cellular localization of β-catenin in HTB75 and HTB161 cells. Treatments with F7 and niraparib+F7 substantially reduced *DVL2* expression in both cell lines (Figure 11a,b), whereas niraparib only treatment reduced *DVL2* expression in HTB161 and increased it in HTB75 (Figure 11a,b). In non-induced HTB161 cells epithelial characteristics of, e.g., smaller cells that formed a tight monolayer, were evident (Figure 11c). In these non-induced cells, β-catenin was distributed in the cell membrane in thickened cell contacts (Figure 11c, white arrows). In contrast, in HTB161 cells induced for mesenchymal phenotype [21] bigger cells that were detached from each other, a loose monolayer and large spaces between cells were observed (Figure 11d, yellow arrows). In these induced cells, β-catenin was mostly absent from cell membrane (Figure 11d). Treatment with niraparib substantially increased the epithelial phenotype and membrane distribution of β-catenin in the induced cell population (Figure 11e), whereas treatment with F7 did that to a much lesser extent (Figure 11f). The combined niraparib+F7 treatment resulted in a marked increase in the epithelial phenotype and membrane distribution of β-catenin in the induced cell population (Figure 11g). 

In the HTB75 cell line, in contrast to epithelial features and β-catenin membrane localization of non-induced cells (Figure 11h), mesenchymal features and a reduced level of β-catenin in the cell membrane were observed in the induced cell population (Figure 11i). Epithelial features and β-catenin membrane localization were re-established, to some extent, mainly with niraparib treatment of the induced cell population (Figure 11j), and, to a lesser extent, with F7 (Figure 11k) and the combined treatment (Figure 11l). 

### 2.11. Determining the Effects of Treatment on an OC Patient’s Cells

**Figure 12:**
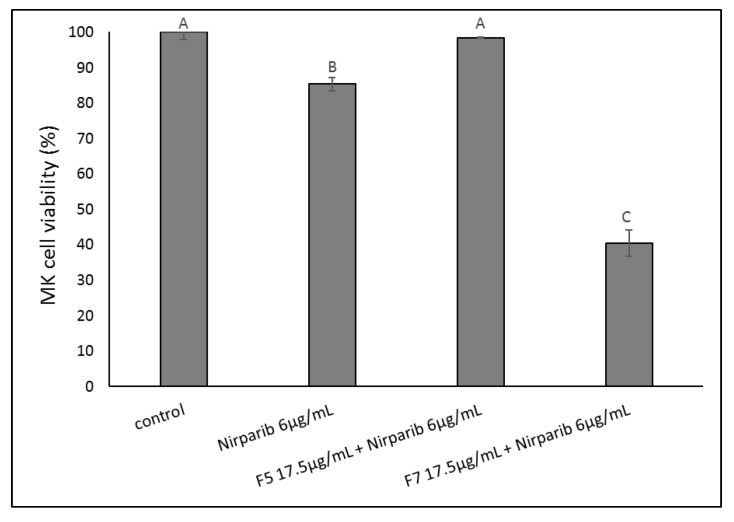
Cell viability of MK cells following treatment with niraparib, niraparib+F5 or niraparib+F7. Cell viability was determined by XTT assay at 48 h as a function of live cell number. Control was vehicle control (1.00% *v*/*v* methanol + 0.60% *v*/*v* DMSO). Error bars indicate ± SE (*n* = 2). Levels with different Upper case letters were significantly different from all combinations of pairs by Tukey–Kramer honest significant difference (HSD; *p* ≤ 0.05).

The cytotoxic activity of the niraparib+F5 and niraparib+F7 synergistic mixtures was assessed on an OC patient’s cells (MK), isolated from a cancerous deep femoral lymph node. These cells showed only low sensitivity to niraparib and no sensitivity to a combination of niraparib and F5 (Figure 12). However, the combined treatment of niraparib+F7 led to substantial cell death (Figure 12). 

## 3. Discussion

We identified two compositions of phytocannabinoid mixtures from the extract of a high-THC cannabis strain with significant cytotoxic activity against OC cell lines, HTB75, HTB161 and HTB76, all high-grade serous ovarian cancer cells [22]. To identify the active compounds, the crude extract was fractionated and active fractions were identified. Similar to other studies [19,20], the active fractions exhibited a cytotoxic advantage over the crude extract. Cytotoxicity was apparent already at 24 h, whereas the IC50 values were reduced with increased treatment time in the examined OC cell.

The fractions contained both phytocannabinoids and terpenes. However, once phytocannabinoid standards were used, in the ratio found in each of the fractions (i.e., F5-SM, F7-SM), cytotoxic activity mostly increased. These results suggest that the active compounds for the cytotoxic activity against OC cell lines are the phytocannabinoids, and the terpenes or other unidentified compounds present in both the crude extract and fractions may interfere with this activity.

The most active fractions were found to be F7 and F5, and their phytocannabinoid composition was determined. Subsequently, the most effective combinations of the main phytocannabinoids [6,8] for cytotoxic activity against OC cells were determined. The combinations of THC and CBC in F7 were the most effective, with or without CBG. In the F5 combinations, THC+CBG was highly active. These combinations were more effective than THC only.

Another fraction that contained THC, DQ-F6 [19], showed cytotoxic activity against OC but to a lesser extent than F5 or F7. F6 contained relatively more THC in relation to CBG than F7, and contained no CBC [19], which may explain its lower activity.

In multiple studies, THC and CBG have shown anticancer activity in vitro and in vivo for a variety of cancer types, and THC has exhibited this also in clinical trials [23]. CBC was shown to have anticancer activity on prostate carcinomas [24]. In addition, we previously found that a combination of THC and CBC was highly active against urothelial carcinoma cells [25]. Together, this study and others (e.g., [19,20,25]), highlight the importance of the appropriate combination of compounds for highly specific anti-cancer activity in vitro; in the case of cytotoxicity to OC cells, a combination of THC and CBC with or without CBG.

In this study, treatment with F5, F7, F5-SM or F7-SM led to an increase in apoptotic cell death in HTB75 and HTB161 cells. In previous studies, phytocannabinoids were shown to induce apoptosis in cancer cells and to inhibit cancer cell proliferation [9,11]. 

Combination with a CB2 inverse agonist significantly blocked the cytotoxic activity of F5, F7 and their corresponding standard mixes in the HTB75 cell line, and mitigated cytotoxicity of F7 in the HTB161 cell line. Notably, CB2 is overexpressed in malignant endometrial carcinoma cells but not in healthy cells [26]. The TRPV2 antagonist reduced the cytotoxic activity of F7 and F7-SM, to some extent, in HTB75 only. TRPV2 is expressed in OC tissues at higher levels than in adjacent normal tissues [27]. Both *CB2* and *TRPV2* genes were found to be expressed in the HTB75 OC cell line, but not to be induced by the fraction treatments. Neither the TRPA1 blocker, the TRPV1 antagonist nor the CB1 inverse agonist reduced activity, suggesting TRPA1, TRPV1 and CB1 are not involved in the cytotoxic activity of these compounds in HTB75 cells. In contrast, CB1 inverse agonist, TRPA1 blocker, TRPV1 antagonist, and TRPV2 antagonist significantly increased the activity of F7 in HTB161, suggesting some involvement of these other receptors in F7 cytotoxic activity in this particular cell line. Notably, the higher level of CB2 and TRPV2 expression in malignant tissues, and their possible engagement with the cannabis fractions and compounds, may position CB2, and possibly TRPV2, receptors as a target for cannabis-based therapy.

A plausible approach for the care of an OC patient is to combine the cannabis-based treatment with chemotherapy. Hence, we examined the effects of cannabis and chemotherapy/monotherapy drugs co-treatment on HTB75 and HTB161 cell viability. Niraparib is a potent Poly (ADP-ribose) polymerase (PARP)-1 and PARP-2 inhibitor. PARPs are a family of nuclear proteins that allocate single-strand breaks in DNA, bind, and activate recruitment of repair factors [28]. These processes are important for cell proliferation. Indeed, PARP inhibition by niraparib leads to cell apoptosis [28]. 

We found that niraparib acts synergistically with F5 and F7 at some of the examined concentrations in HTB75. Synergy between F7 and niraparib was evident on the HTB161 cell line as well. Synergy between the standard mixes F5-SM or F7-SM and niraparib was at a lower level in comparison to that of niraparib and plant fractions. Gemcitabine did not show substantial synergistic activity with the cannabis-based treatments. The fact that the plant fractions, containing both phytocannabinoids and terpenes, acted synergistically with the niraparib, while the phytocannabinoids alone exhibited reduced level of synergy, suggests that terpenes or other compounds present in the fractions facilitate this synergy and might even increase cell sensitivity to niraparib. 

Moreover, the mixture of niraparib+F5 or niraparib+F7 at the synergistic ratio was ~50-fold more cytotoxic to HTB75 cells than to normal keratinocytes (HaCaT cells), once examined under complete confluence (complete confluence conditions were used to reduce non-cancerous cell proliferation in the control treatment). The low cytotoxicity to normal cells suggests that treatment with F5 or F7 in combination with niraparib could prove to be an effective, and, at least to some extent, cancer-specific treatment. The activity on an OC patient’s cells isolated from a cancerous deep femoral lymph node by the combination of niraparib+F7 supported this suggestion. Treatment of OC patients by niraparib was examined in clinical trials [29] and the EU and FDA approved olaparib to be used in combination with bevacizumab to treat advanced ovarian cancer; olaparib, similarly to niraparib, is a PARP inhibitor [30,31]. 

Since aberrant activation of the canonical Wnt pathway plays a critical role in OC development [15], and in epithelial ovarian cancer FZD1 expression is increased [18], we examined Wnt pathway gene expression following F7 and/or niraparib treatments of the two examined cell lines (HTB75 and HTB161). Our findings suggested that F7, and, in some cases, niraparib or niraparib+F7 treatments, significantly reduced *FZD4* and *FZD1* expression in both HTB161 and HTB75 cells. Additionally, treatment with F7 increased *DKK1* expression in HTB161. DKK1 inhibits the dimerization of FZD and LRP5/6 receptor and directly prevents FZD activation; it is downregulated in OC tumors [15]. The expression of *Wnt* was slightly reduced (for *Wnt10B*) or induced (for *Wnt5A*) by the treatment in HTB161, but reduced in HTB75. Importantly, F7 co-treatment with CB2 IA diminished the alterations in *FZD* gene expression in both HTB75 and HTB161 cell lines, further elucidating the involvement of CB2 in the effect of F7 on OC cells and on Wnt-pathway related gene expression. In line with our results, it was previously demonstrated that CBD, the 2,3-epoxy derivative of CBD and cannabidivarin (CBDV) exhibited, in a dose-dependent manner, considerable inhibitory activity against the Wnt/β-catenin pathway, based on analysis of TCF-dependent β-catenin mediated transcription activity [32]. 

Canonical Wnt signaling facilitates EMT [17]. Increased protein levels of DVL2 enhance canonical Wnt signaling and EMT [33]. In ovarian cancer, cyclin G2 repressed the Wnt/β-catenin signaling pathway by downregulating key Wnt components, including DVL2 [34]. Moreover, DVL2 is suggested to play a part in epithelial OC progression and might be an independent prognostic factor and a prospective therapeutic target in this disease [35]. Treatment of cells with F7 or niraparib+F7 substantially reduced *DVL2* expression in both cell lines. Moreover, treatment of cells induced for mesenchymal phenotype [21] with niraparib+F7 led to re-acquisition of the epithelial phenotype and a substantial re-distribution of β-catenin to the cell membrane (in HTB161 to a greater extent than HTB75), further solidifying the effect of the niraparib and F7 treatments on the canonical Wnt pathway signaling. Modulation of the Wnt signaling pathway leads to cell apoptosis in many cases [36], as in the case of treatments with F7 and niraparib. Hence, niraparib and F7 cytotoxic activity might be mediated, at least partially, via modulation of this signaling pathway.

**Figure 13:**
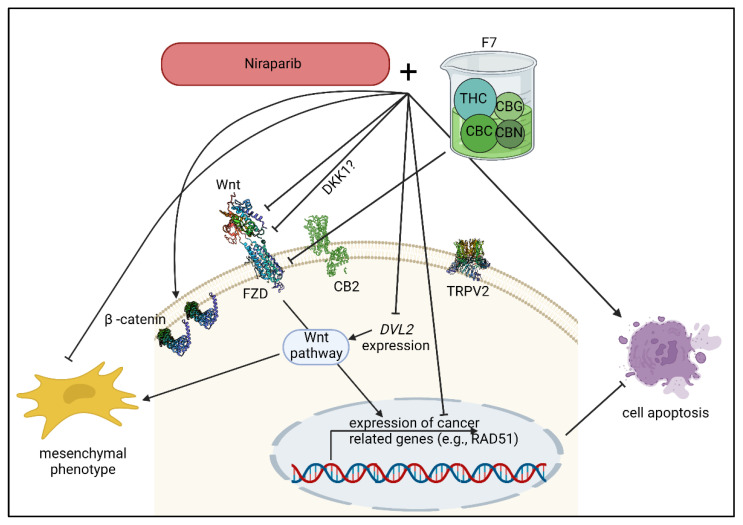
A suggested, simplified model for F7 and niraparib activity in OC cells in relation to the Wnt pathway. F7, niraparib or niraparib+F7 treatments significantly reduced *FZD4* and *FZD1* expression. This activity for F7 was partially dependent on CB2 activation. The expression of *Wnt* was altered by the treatments. Treatment with F7 also increased *DKK1* expression in one of the examined cell lines. DKK1 inhibited the dimerization of FZD (with LRP5/6 receptor, not shown), directly preventing FZD activation. As a possible result of the alterations of the canonical Wnt signaling pathway, mesenchymal phenotype was inhibited, *DVL2* and *RAD51* expression was reduced and cell apoptosis was induced. Wnt, wingless/int1; FZD, Frizzled; DKK, Dickkopf; DVL2, Dishevelled2; RAD51, recombinase Rad51; THC, Δ9–tetrahydrocannabinol; CBC, cannabichromene; CBG, cannabigerol; CBN, cannabinol. Created with BioRender.com (accessed on 12 October 2022).

Notably, members of the PARP family are positive regulators of Wnt/β-catenin signaling in OC and the activation of the Wnt/β-catenin signaling was previously shown to be involved in inducing resistance to the PARP inhibitor olaparib [15]. A PARP1 inhibitor was shown to inhibit β-catenin signaling in HeLa (cervix adenocarcinoma) and SiHa (cervix squamous cell carcinoma) cells [37]. Moreover, it was shown that in Wnt-addicted cancers, Wnt inhibition synergized with the PARP inhibitor olaparib [16]. It was suggested that Wnt inhibition created a BRCA-like state that sensitizes cancer cells to DNA damaging agents in [16]. Since neither HTB161 nor HTB75 bear BRCA mutations, it might be that at least part of the mechanism behind the niraparib and F7 synergy involves alterations to Wnt signaling (Figure 13). This suggestion was fortified by the finding that *RAD51* expression decreased, most notably in HTB161, with the co-treatments. RAD51 is a member of the Fanconi anemia repair pathway, and is dependent, in Wnt-high cancers, on Wnt/β-catenin signaling [16]. Nevertheless, differences in *RAD51* and Wnt signaling-pathway gene expression were recorded between HTB161 and HTB75, suggesting some variance between cell lines in the molecular mechanisms that underlie the treatments’ effects. 

## 4. Materials and Methods

### 4.1. Plant Extraction

The dry inflorescence of the high Δ9–tetrahydrocannabinol (THC) *C. sativa* strain Dairy Queen (DQ) (IMC, Petah Tikva, Israel) was extracted, as described previously [19,20]. Briefly, decarboxylation was carried out by heating the dry extract to 220 °C for 10 min. The heated extract was dissolved in methanol and filtered through a 0.45 µm syringe filter. Following evaporation, the weighted extract was re-dissolved in methanol to the desired concentration.

### 4.2. Extract Fractionation

Extract fractionation was done, as described previously [19,20]. Briefly, a flash chromatography apparatus (Flash Pure, Buchi, C-810), equipped with a diode array detector, was used to fractionate the decarboxylated crude extract. An Ecoflex C-18 80 g, 50 µm spherical, maximum pressure 180 psi, column was used for separation, with 80–85% methanol in water as the mobile phase. The flow rate was 30 mL/min. The organic solvent (methanol) of each fraction was separately removed by using a rotary vacuum evaporator at 30 °C. The remaining aqueous phase, containing the compound of interest, was lyophilized to obtain a dried powder. Each dried fraction tube was weighed separately and reconstituted with methanol to produce a 1 or 2 mg/mL solution; for IC50 experiments, a 5 mg/mL solution was used. All solutions were stored at −20 °C.

### 4.3. Chemical Analysis

Chemical analysis was done, as described previously [19,20]. Briefly, high performance liquid chromatography (HPLC, 1260 Infinity II, Agilent), equipped with a Raptor ARC-18 for LC-UV column (150 mm × 4.6 mm ID, pore size 2.7 µm), was used for chemical analysis. A gas chromatography-mass spectrometer (GC/MS; GC8860-MS5977B Agilent), equipped with a 30 m, 0.25 mm ID, 5% cross-linked phenylmethyl siloxane capillary column (HP-5MS) with 0.25-μm film thickness, was used for chemical analysis, as described in [20]. An amount of 10 μL of each sample fraction was transferred into GC vials with an insert, dried under a gentle stream of nitrogen and dissolved in 100 μL of hexane. Sample volume for injection was 1 μL. Helium was used as the carrier gas at a constant flow of 1.1 mL s^−1^. An isothermal hold at 50 °C was maintained for 2 min, followed by a heating gradient of 6 °C min^−1^ to 300 °C, with the final temperature held for 4 min. Peaks were assigned using spectral libraries (NIST 14.0 and 17.0) and compared with MS data obtained from the injection of standards purchased from LGC Standards.

### 4.4. Standard/Material Preparation and Use

Standard/Material preparation and use was done, as described previously [19,20]. Briefly, the cannabinoid standards at a concentration of 1 mg/mL in methanol used in this study included THC (34067; Restek, Bellefonte, PA, USA), CBC (34092; Restek), CBG (34091; Restek) and CBN (34010; Restek). Inverse agonists (IA) to CB1 and CB2 were AM251 (ab120088; Abcam, Cambridge, UK) and SR144528 (ab146185; Abcam), respectively. The TRPA1 blocker used was HC-030031 (ab120554; Abcam). TRPV1 and TRPV2 antagonists were ab141772 (Abcam) and Tranilast 1098/10 (Abcam), respectively. All IAs, the blocker and antagonists were dissolved in dimethyl sulfoxide (DMSO) at a concentration of 10 mM and used for cell treatment at a final concentration of 10 µM. Chemotherapy solutions for synergy tests included niraparib (AG0038ZU; Angene, Nanjing, China) and gemcitabine (461060010; Acros Organics, Beijing, China) both dissolved in DMSO. Solutions for induction of malignant features [21] included IL-1β (200-01B; PeproTech, Cranbury, NJ, USA) and TNFα (300-01A; PeproTech) both dissolved in pure water. Solvents (methanol and/or DMSO) were used as a negative control and niraparib was used as a positive control in all the biological assays. The control in any given experiment was set at a concentration to match the vehicle concentration in the highest concentration treatment. 

### 4.5. Cell Culture

OC cell lines; HTB75 (ATCC, CAOV-3; Adenocarcinoma), HTB161 (ATCC, OVCAR-3; Adenocarcinoma) and HTB76 (ATCC, CAOV-4; Adenocarcinoma) cell lines were cultured in Dulbecco’s Modified Eagle Medium (DMEM) (01-055-1A; Biological Industries, Beit HaEmek, Israel) supplemented with 10% fetal bovine serum (FBS) (04-127-1A; Biological Industries), RPMI medium (01-100-1A; Biological Industries), supplemented with 20% FBS, and Leibovitz’s L-15 Medium (01-115-1A; Biological Industries) supplemented with 20% FBS respectively. HaCaT (Keratinocytes, CLS Cell Lines Service GmbH, Eppelheim, Germany; [21]) skin cells were cultured in DMEM supplemented with 10% FBS. All media were supplemented with 1% Pen-Strep, 1% L-Glutamine and 0.02% plasmocin. Cells were incubated at 37 °C in a humidified atmosphere; HTB75, HTB161 and HaCaT were grown in an environment containing 5% CO_2_ and 95% air, while HTB76 cells were grown in air. Induction of the mesenchymal phenotype was done as described in [21], with modifications: HTB75 and HTB161 were seeded in complete medium 3 days prior to induction. Induction conditions for HTB161 were RPMI medium, including FBS 5% and IL-1β 30 ng/mL, and for HTB75 DMEM medium, including FBS 1%, IL-1β 30 ng/mL and TNFα 100 ng/mL for 48 h. Treatments were applied for 16 h, while maintaining induction conditions. 

### 4.6. Isolation of OC Cells from Patient MK

Samples of proliferating cells containing approximately 3M cells were received from the Ridley-Tree Cancer Center (Santa Barbara, CA, USA)/SEngine (Seattle, WA, USA), Sample ID SE0538, CLIA Number: 50D2106197, Quorum Review Institutional Review Board approved 19 June 2018, WA State Medical Test: MTSC.FS.60622201. Cells originated in a single cancerous deep femoral lymph node, removed via surgical resection from OC patient, MK, by a gynecological oncology surgeon, approximately five years after her primary diagnosis. The cells were cultured for personalized diagnosis by SEngine Precision Medicine [38,39]. Cells were propagated using DMEM supplemented with 10% FBS, 1% Pen-Strep, 1% L-Glutamine and 0.02% plasmocin. 

New generation sequencing (NGS) and immunohistochemistry (IHC) profiling and analysis was carried out on the cancerous lymph node tissue by Foundation Medicine, Inc. (Cambridge, MA, USA), using their FoundationOne CDx protocol, targeting 324 cancer-related genes. In this analysis cells showed mutant TP53 C135Y and STK11 splice site 735-1G>C, but all other genes tested (including BRCA1 and BRCA2) lacked cancer-associated mutations. A clinical IHC indicated that cancer was strongly positive for PAX8 and WT1. The patient signed the informed consent agreeing to the use of tissue samples for research purposes.

### 4.7. Cell Viability Assay

The cell viability assay was done as described previously [19,25]. Briefly, cells were seeded into 96-well plates at a density of 1 × 10^4^ per well (in 50 µL/well) in complete medium and incubated at 37 °C overnight to allow attachment. The following day, cells were treated in triplicate with plant extracts, fractions, or cannabinoid standards at a volume of 50 µL/well at different concentrations, as described in each experiment. In experiments where CB1 or CB2 inverse agonists (AM251 and SR144528, respectively), TRPV1 or TRPV2 antagonists (SB-366791 and Tranilast, respectively), or the TRPA1 blocker (HC-030031) were used, they were added along with the treatment at concentration of 10 µM. Treated cells were incubated for 24, 48 or 72 h at 37 °C. Subsequently, XTT reagents (2,3,-bis(2-methoxy-4-nitro-5-sulfophenyl)-5-[(phenylamino)-carbonyl]-2H-tetrazolium inner salt) (20-300-1000, Biological Industries, Israel) were added to the cells for 2 h at 37 °C in a humidified 5% CO_2_–95% air atmosphere. Absorbance was recorded using a Synergy H1 hybrid reader photometer (BioTek) at 490 nm with a 650 nm reference wavelength. Cell viability was estimated using the equation: %Cell Viability=100×((A490−A650) of treatment÷(A490−A650) of solvent control). A490 and A650 were the absorbencies of the XTT colorimetric reaction. Absorbance of the media alone (blank) was subtracted from the readings. For dose response assay, GraphPad Prism version 6.1 (https://www.graphpad.com/scientific-software/prism/ (accessed on 28 October 2021), GraphPad Software Inc., San Diego, CA, USA) was employed to produce dose-response curves (data points were connected by non-linear regression lines of the sigmoidal dose-response inhibition curve relation) and determination of IC50 values. For assay of full confluence, cells were seeded in 96-well plates at a density of 1 × 10^4^ per well (at 100 µL/well) in complete medium and were incubated at 37 °C for 5 days to allow 100% confluence.

### 4.8. Flow Cytometry Analysis

Flow cytometry analysis was done, as described previously [19,25]. For the apoptosis assay 5 × 10^5^ HTB75 or HTB161 cells were seeded in 2 mL medium per well, 24 h before treatment in 6-well TC plates. The treatment period was 48 h, and cells were harvested after applying 250 μL trypsin for 5 min, adding a complete medium to neutralize the trypsin, and centrifuging for 5 min at 1600 rpm. Cell pellets were resuspended and washed twice with 1 mL of phosphate buffered saline (PBS). Cells were assessed using an MEBCYTO Apoptosis Kit with Annexin V-FITC and PI (4700; MBL, Woods Hole, MA, USA). Staining was carried out according to the manufacturer’s instructions. The cells in each sample were resuspended in 85 μL of Annexin binding buffer. Cells were stained with 10 μL of Annexin V- FITC solution and 5 μL of propidium iodide (PI) working solution, followed by incubation in the dark at room temperature for 15 min. Next, 400 μL of Annexin V binding buffer was added to each tube. Rates of apoptosis were analyzed with flow cytometry, LSR-FORTESSA (BD, Franklin Lakes, NJ, USA). Cells were considered apoptotic if they were Annexin V+/PI− (early apoptosis) or Annexin V+/PI+ (late apoptosis). Live cells were defined as Annexin V−/PI−, and Annexin V−/PI+ as necrotic. 

### 4.9. Staining and Confocal Microscopy

Cells (1 × 10^4^ cells/plate) were seeded in a 35 mm glass bottom cell culture dish in 500 µL complete medium and incubated at 37 °C. Three days later, cells were induced for stress with IL-1β for HTB161 and with IL-1β+TNFα for HTB75. Treatments were given after 48 h of induction, for 16 h at sub-lethal concentrations. For fixation and permeabilization of cells, Transcription Factor Staining Buffer Set (130-122-981; Miltenyi Biotech, Bergisch Gladbach, Germany) was used, and cells were incubated for 30 min at 4–8 °C. For staining, β-Catenin monoclonal antibody (130-121-990-PE; REAfinity™ Miltenyi Biotech, Germany) was used, and cells were incubated for 40 min at 4–8 °C. Hoechst (EasyProbes™ 33342; ABP Bioscience, Beltsville, MD, USA) was used for nuclear staining. Cell microscopy and image acquisition was carried out using a Leica SP8 laser scanning microscope (Leica, Wetzlar, Germany), equipped with 405 and 552 nm solid state lasers, HC PL APO CS 63×/1.2 water immersion objectives (Leica, Wetzlar, Germany) and Leica Application Suite X software (LASX, Leica, Wetzlar, Germany). Hoechst, 5(6)-Carboxyfluorescein and PE (red)emission signals were detected with PMT and HyD (hybrid) detectors in ranges of 415–490 nm, and 565–660 nm, respectively.

### 4.10. Analysis of Combined Drug Effects

Drug synergy was determined from XTT results by the Bliss independence drug interaction model, as described in [20,25], on HTB75 and HTB161 cells, defined by the following equation: Exy = Ex + Ey – (ExEy), where (Exy) is the additive effect of the drugs x and y as predicted by their individual effects (Ex and Ey). The synergy expressed by the delta of EXy and Ex or Ey and the values differed on a scale between 0 (no synergy) to 1 (high synergy). Negative values denoted an inverse effect.

### 4.11. Quantitative Real-Time PCR

Determination of gene expression was done, as described previously [19,25]. Cells were seeded in a 6-well plate at a concentration of 1.5 × 10^6^ cells in 3 mL medium per well. After 24 h incubation, cells were treated with cannabis compounds and/or chemotherapy drugs for 3, 6 or 9 h. Cells were harvested, and RNA was extracted using TRI reagent (Merck). RNA was reverse-transcribed in a total volume of 20 μL (PB30.11-10; qPCRBIO, PA, USA) according to the manufacturer’s protocol. PCR was performed in triplicate using a qPCR SyGreen Blue Mix (PB20.16-20; qPCRBIO) and StepOne Plus system (Applied Biosystems Thermo Fisher Scientific, Waltham, MA, USA). The expression of each target gene was normalized to the expression of HPRT mRNA following the 2^−ΔΔCt^ method presenting the differences (∆) in threshold cycle (Ct) between the target gene and HPRT gene, using the following equation: ΔΔCt= ΔCt treatment−ΔCt control. Experiments were repeated three times. The primers included were those for *CB2* (CNR2, Gene ID: 1269) (forward) 5′-ATCATGTGGGTCCTCTCAGC-3′ and (reverse) 5′-GATTCCGGAAAAGAGGAAGG-3′; *TRPV2* (Gene ID: 51393) (forward) 5′-TGCTCACCTACATCCTGCTG-3′ and (reverse) 5′-GCACCACCAATAGCCATTCT-3′; *FZD1* (Gene ID: 8321) (forward) 5′-GTGAGCCGACCAAGGTGTAT-3′ and (reverse) 5′-AGCCGGACAAGAAGATGATG-3′; *FZD4* (Gene ID: 8322) (forward) 5′-CCTGGCCAGAGAGTCTGAAC-3′ and (reverse) 5′-TTGGTTCCCACAGAGTGACA-3′; *DKK1* (Gene ID: 22943) (forward) 5′-CATCAGACTGTGCCTCAGGA-3′ and (reverse) 5′-CCACAGTAACAACGCTGGAA-3′; *Wnt5A* (Gene ID: 7474) (forward) 5′-CAAGGGCTCCTACGAGAGTG-3′ and (reverse) 5′-CTTCTCCTTCAGGGCATCAC-3′; *Wnt10B* (Gene ID: 7480) (forward) 5′-CTGGTGCTGCTATGTGCTGT-3′ and (reverse) 5′-ATCAGAGCAAAGGGCTGAAA-3′; *RAD51* (Gene ID: 5888) (forward) 5′-TCACGGTTAGAGCAGTGTGG-3′ and (reverse) 5′-GTGGTGAAACCCATTGGAAC-3′; *DVL2* (Gene ID: 1856) (forward) 5′-CATGAGCAACGATGACGCTG-3′ and (reverse) 5′-AGGGTCAATTGGCTGGATGG.

### 4.12. Statistical Analysis

All results presented were mean ± standard error (SE) of replicate analyses and were either representative of, or included, at least three independent experiments, except for determination of viability of MK cells, where *n* = 2. Means of replicates were subjected to the Tukey–Kramer test or Student’s t-test using the JMP statistical package (https://www.jmp.com/en_us/home.html (accessed on 28 February 2022), SAS Inc., Cary, NC, USA) and considered significant when *p* ≤ 0.05 [19,25].

## 5. Conclusions

We identified cannabis compounds with substantial cytotoxic activity against OC cells in vitro, which involved apoptosis. This activity was found to be considerably stronger on cancer cells than on normal cells. Indeed, CB2 and TRPV2, which might be involved with F7 activity, are expressed more often in malignant tissue [26,27]. Hence, cannabis-based therapies targeting cancer cells with reduced effect on the healthy surrounding tissues may be possible. 

Moreover, we identified synergistic activity between the cannabis extract fractions and niraparib in vitro. We found that treatments by F7 and/or niraparib led to alterations in the Wnt signaling pathway. These alterations included reduction of *FZD* expression by F7 or niraparib+F7 treatments; in the case of F7, its activity was at least partially mediated via CB2. They also induced alterations in other Wnt signaling-pathway related gene expression, reduction of *DVL2* and *RAD51* expression (the latter mainly in HTB161) and inhibition (in both cell lines, but in HTB161 to a greater extent) of the mesenchymal phenotype and re-distribution of β-catenin to the cell membrane. Eventually, F7 and/or niraparib led to induction of cell apoptosis (Figure 13). However, additional studies should be performed to fully characterize the molecular mechanisms that underlie F7 anti-OC activity and niraparib and F7 synergy; this is especially important in light of the differences in gene expression between the examined cell lines. The differences between cell lines may result from their genetic differences. For example, HTB161 and HTB75 are different is some aspects related to the canonical Wnt pathway [40,41].

We suggest that cannabis might be regarded as a complementary and effective anti-cancer treatment for OC. Given the favorable safety profile of phytocannabinoids, compared to standard pharmacotherapies (e.g., [42]), we propose that clinical trials with cannabis-based products are desperately needed for OC patients.

## Figures and Tables

**Table 1:** Quantitative PCR determination of the RNA steady state level in the HTB75 cell line of CB2 receptor (*CNR2*) or *TRPV2* genes, after treatment with F5 or F7 for 6 h relative to control.

Treatment	CB2 Expression (Mean ± SE)	TRPV2 Expression (Mean ± SE)
Control	1.00 ± 0.12 ^A^	1.00 ± 0.09 ^a^
F5 17.5 μg/mL	1.06 ± 0.11 ^A^	1.15 ± 0.11 ^a^
F7 17.5 μg/mL	1.11 ± 0.10 ^A^	1.15 ± 0.13 ^a^

Control was the vehicle control (1% *v*/*v* methanol). Gene transcript values were determined by quantitative PCR as a difference between the target gene versus a reference gene (HPRT). Values were calculated relative to the average expression of target genes in treated versus control using the 2^−ΔΔCt^ method. Levels with different Upper or lower case letters with the same font and style were significantly different from all combinations of pairs by Tukey–Kramer honest significant difference (HSD; *p* ≤ 0.05; *n* = 3).

**Table 2:** Quantitative PCR determination of the RNA steady state level in HTB161 and HTB75 cell lines of *RAD51* gene, after treatment with F7 and/or niraparib for 6 and 9 h relative to control.

	*RAD51* Expression in HTB161 (Mean ± SE)		*RAD51* Expression in HTB75 (Mean ± SE)	
Duration of Treatment	6 h	9 h	6 h	9 h
Control	1.00 ± 0.04 ^A^	1.00 ± 0.00 ^a^	1.00 ± 0.10 ^A^	1.01 ± 0.12 ^a^
F7	0.54 ± 0.01 ^B^	0.73 ± 0.05 ^b^	0.67 ± 0.04 ^A^	0.89 ± 0.05 ^a^
Niraparib	0.63 ± 0.02 ^B^	0.59 ± 0.02 ^bc^	1.30 ± 0.24 ^A^	1.02 ± 0.16 ^a^
Niraparib+F7	0.65 ± 0.08 ^B^	0.55 ± 0.03 ^c^	0.83 ± 0.09 ^A^	1.15 ± 0.04 ^a^

Control was the vehicle control (1% *v*/*v* methanol + 0.6% *v*/*v* DMSO). Concentrations used: for HTB75, niraparib 6 µg/mL, F7 17.5 µg/mL; for HTB161, niraparib 25 µg/mL, F7 20 µg/mL. Gene transcript values were determined by quantitative PCR as a difference between the target gene versus a reference gene (HPRT). Values were calculated relative to the average expression of target genes in the treated versus control using the 2^−ΔΔCt^ method. Levels with different Upper or lower case letters with the same font and style were significantly different from all combinations of pairs by Tukey-Kramer honest significant difference (HSD; *p* ≤ 0.05; *n* = 3).

## Data Availability

Data is contained within the article and Supplementary Material.

## Supplementary Materials

The following supporting information can be downloaded at: https://www.mdpi.com/article/10.3390/molecules27217523/s1, Figure S1. Cell viability of HTB76 cells following treatment with *C. sativa* DQ fraction F5 (a), F7 (b), F5-SM (c) and F7-SM (d) at different concentrations. The IC50 values were calculated from 5P logistic curve fit using GraphPad Prism version 6.1. Error bars indicate ± SE (*n* = 3). Concentration 0 was a vehicle-treated control (3.50% *v*/*v* methanol); Supplementary Figure S2. Cell viability of HTB161 cells following treatment with F7, with or without CB1 and CB2 inverse agonists (IA), a TRPA1 blocker (B), and TRPV1 or TRPV2 antagonists (AN) for 48 h. Cells were treated with F7 (24.46 µg/mL), with or without the receptor IA, B or AN (10 µM) (a). The effect of IA, B or NA on cell viability (b). Cell viability was determined by XTT assay as a function of live cell number. Niraparib (24.75 µg/mL) served as a positive control. Control is vehicle control (1.23% *v*/*v* methanol + 1.00% DMSO). Error bars indicate ± SE (*n* = 3). Levels with different Upper case letters are significantly different from all combinations of pairs according to the Tukey–Kramer honest significant difference (HSD; *p* ≤ 0.05). Supplementary Figure S3. Synergistic interactions between F5, F7, F5-SM or F7-SM with gemcitabine on cell viability of HTB75 cells following combined treatments. Control was the vehicle control (1.75% *v*/*v* methanol + 0.05% *v*/*v* DMSO). Synergy of cytotoxic activity calculated based on the Bliss independence drug interaction model. Synergy is apparent when the experimental (observed) value of cell survival inhibition is higher than the calculated (expected) value. Values of delta of observed minus expected are shown in the *Y* axis. In Supplementary Table S6 were the different letters that signify different levels of the delta values from all combinations of pairs according to the Tukey–Kramer honest significant difference test (*n* = 3; HSD; *p* ≤ 0.05), for the synergistic delta values of F5, F7, F5-SM or F7-SM with gemcitabine; Supplementary Table S1. The F7 composition of terpenes; Supplementary Table S2. Summary of IC50 values determined at different time points of treatments at the 3 cell lines (HTB75, HTB161 and HTB76); Supplementary Table S3. Treatment layout with THC, CBG, CBC and CBN standards on the HTB75 cell line (µg/mL); Supplementary Table S4. Different Upper case letters signify delta values that were significantly different from all combinations of pairs according to Tukey–Kramer honest significant difference (HSD; *p* ≤ 0.05). Delta values calculated according to the Bliss model between the experimental (observed) and the calculated (expected) values of the synergistic interactions between F5, F7, F5-SM or F7-SM and niraparib on cell viability of HTB75 cells following combined treatments. Delta values are graphically presented in Figure 7; Supplementary Table S5. Different Upper case letters signify delta values that were significantly different from all combinations of pairs according to Tukey–Kramer honest significant difference (HSD; *p* ≤ 0.05). Delta values calculated according to Bliss model between the experimental (observed) and the calculated (expected) values of the synergistic interactions between F7 and niraparib on cell viability of HTB161 cells following combined treatments. Delta values are graphically presented in Figure 7; Supplementary Table S6. Different Upper case letters signify delta values that were significantly different from all combinations of pairs according to Tukey–Kramer honest significant difference (HSD; *p* ≤ 0.05). Delta values calculated according to Bliss model between the experimental (observed) and the calculated (expected) values of the synergistic interactions between F5, F7, F5-SM or F7-SM and gemcitabine on cell viability of HTB75 cells following combined treatments. Delta values are graphically presented in Supplementary Figure S3.

## Sample Availability

Samples of the compounds are available from the authors.
