# Supplementary material for: Phytocannabinoid Compositions from Cannabis Act Synergistically with PARP1 Inhibitor against Ovarian Cancer Cells In Vitro and Affect the Wnt Signaling Pathway
Source: Molecules. 2022 Nov 3;27(21):7523. doi: 10.3390/molecules27217523 (PMC9653955; doi:10.3390/molecules27217523)
Supplement: Supplementary file 1 [file molecules-27-07523-s001.zip › molecules-1995318-supplementary.pdf]

Supplementary Data

Phytocannabinoid compositions from cannabis act synergistically with PARP1 inhibitor against ovarian cancer cells in vitro and affect the Wnt signaling pathway

Nurit Shalev[1,2], Michelle Kendall[3], Seegehalli M Anil[2], Sudeep Tiwari[2], Hadar Peeri[1,2], Navin Kumar[2], Eduard Blausov[1], Ajjampura C Vinayaka[2] and Hinanit Koltai[2,*]

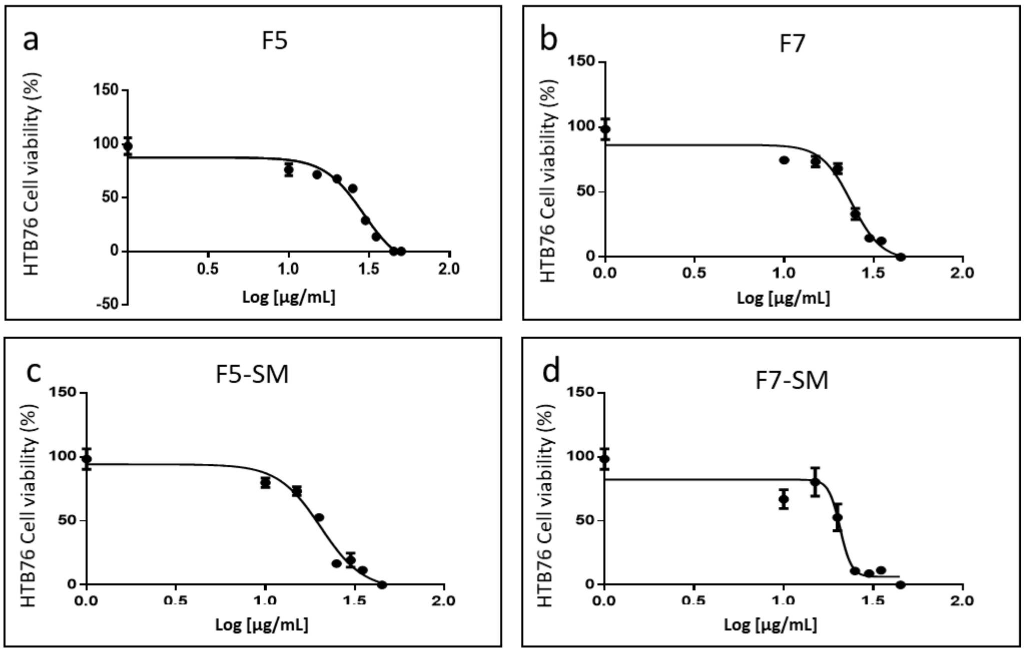

Figure S1. Cell viability of HTB76 cells following treatment with *C. sativa* DQ fraction F5 (a), F7 (b), F5-SM (c) and F7-SM (d) at different concentrations. The IC50 values were calculated from 5P logistic curve fit using GraphPad Prism version 6.1. Error bars indicate ± SE (*n*=3). Concentration 0 is a vehicle-treated control (3.50% v/v methanol).

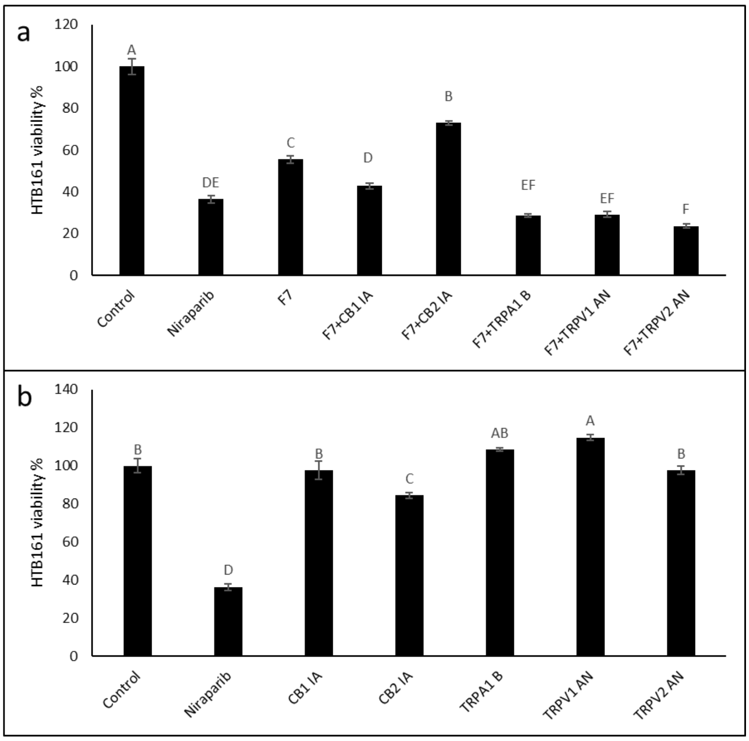

Figure S2. Cell viability of HTB161 cells following treatment with F7, with or without CB1 and CB2 inverse agonists (IA), a TRPA1 blocker (B), and TRPV1 or TRPV2 antagonists (AN) for 48 h. Cells were treated with F7 (24.46 µg/mL), with or without the receptor IA, B or AN (10 µM) (a). The effect of IA, B or NA on cell viability (b). Cell viability was determined by XTT assay as a function of live cell number. Niraparib (24.75 µg/mL) served as a positive control. Control is vehicle control (1.23% v/v methanol + 1.00% DMSO). Error bars indicate ± SE (n=3). Levels with different Upper case letters are significantly different from all combinations of pairs according to the Tukey–Kramer honest significant difference (HSD; $P \leq 0.05$).

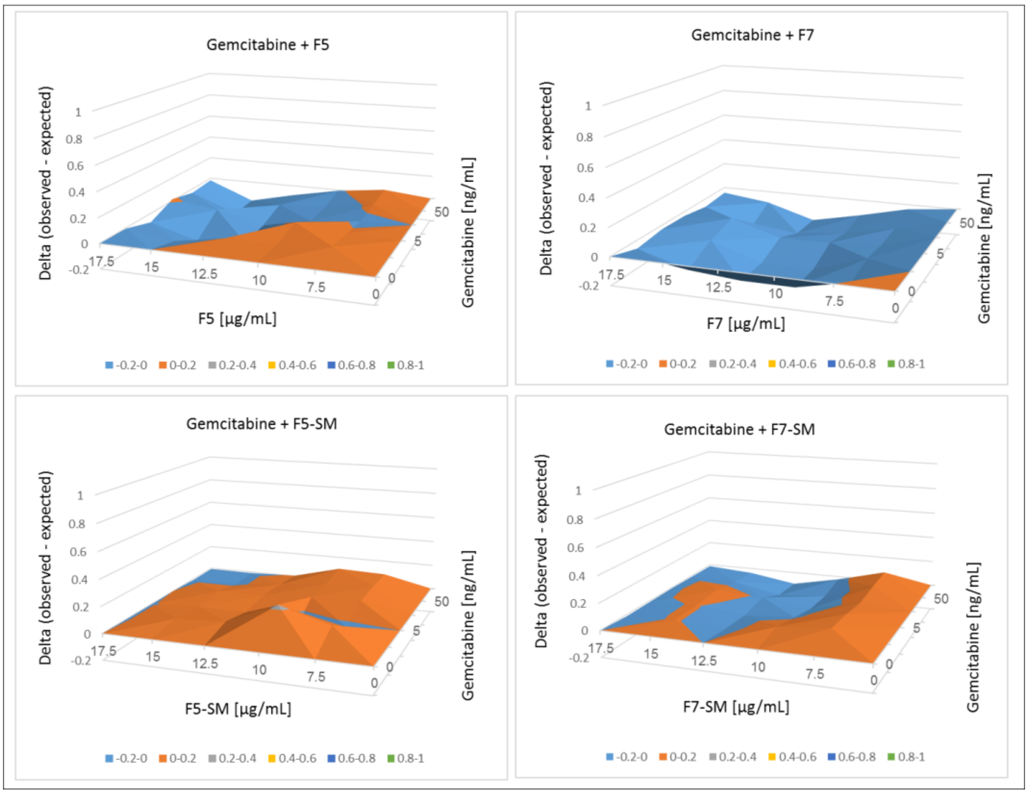

Figure S3. Synergistic interactions between F5, F7, F5-SM or F7-SM with gemcitabine on cell viability of HTB75 cells following combined treatments. Control is the vehicle control (1.75% v/v methanol + 0.05% v/v DMSO). Synergy of cytotoxic activity calculated based on the Bliss independence drug interaction model. Synergy is apparent when the experimental (observed) value of cell survival inhibition is higher than the calculated (expected) value. Values of delta of observed minus expected are shown in the Y axis. In Supplementary Table S6 are the different Upper case letters that signify different levels of the delta values from all combinations of pairs according to the Tukey–Kramer honest significant difference test (*n*=3; HSD; P ≤ 0.05), for the synergistic delta values of F5, F7, F5-SM or F7-SM with gemcitabine.

Table S1. The F7 composition of terpenes.

Compound	% of total compounds in F7
D-Limonene	0.37
α Bergamotene	3.62
Caryophyllene	62.64
(-)-Guaia-6,9-diene	0.59
γ Gurjunene	0.46
γ Muurolene	1.90
onaphthalene	2.24
α selinene	1.86
β selinene	5.29
Bicyclogermacrene	2.26
Cadina-1(10),4-diene	0.51
α Guaiene	1.15
β Guaiene	6.08
Alloaromadendrene	1.28

Selina-3,7(11)-diene	7.92
β maaliene	1.85

Table S2. Summary of IC50 values determined at different time points of treatments at the 3 cell lines (HTB75, HTB161 and HTB76).

	HTB75 IC50 values (μg/mL)					
Incubation time (h)/treatment	DQ	F5	F7	F5-SM	F7-SM	Niraparib
24		18.90	17.17			6.00
48	21.51	18.36	16.95	14.67	13.56	5.10
72		14.31	13.70			3.67

	HTB161 IC50 values (μg/mL)				
Incubation time (h)/treatment	F5	F7	F5-SM	F7-SM	Niraparib
24	37.87	30.46			37.53
48	26.26	24.46	25.11	25.09	24.75
72	25.34	17.66			18.00

	HTB76 IC50 values (μg/mL)				
Incubation time (h)/treatment	F5	F7	F5-SM	F7-SM	Niraparib
48	29.12	23.79	20.38	20.66	27.83

Table S3. Treatment layout with THC, CBG, CBC and CBN standards on the HTB75 cell line (μg/mL).

	F7							F5		
THC	13.0	9.8	12.8	12.8	9.7	9.7	12.6	12.4	12.5	11.9
CBC		3.2			3.2	3.2				
CBN			0.2		0.2		0.2	0.6		0.6
CBG				0.2		0.1	0.2		0.5	0.5
Total μg/mL	13.0	13.0	13.0	13.0	13.0	13.0	13.0	13.0	13.0	13.0
THC	15.0	11.3	14.8	14.8	11.1	11.2	14.5	14.3	14.4	13.8
CBC		3.7			3.7	3.7				
CBN			0.2		0.2		0.2	0.7		0.6
CBG				0.2		0.2	0.2		0.6	0.6
Total μg/mL	15.0	15.0	15.0	15.0	15.0	15.0	15.0	15.0	15.0	15.0

Table S4. Different Upper case letters signify delta values that are significantly different from all combinations of pairs according to Tukey–Kramer honest significant difference (HSD; P ≤ 0.05). Delta values calculated according to the Bliss model between the experimental (observed) and the calculated (expected) values of the synergistic interactions between F5, F7, F5-SM or F7-SM and niraparib on cell viability of HTB75 cells following combined treatments. Delta values are graphically presented in Figure 7.

Niraparib [μg/mL]	F5 [μg/mL]					
	0	7.5	10	12.5	15	17.5
0	FGH	FGH	FGH	FGH	FGH	FGH
1	FGH	DEFG	FGH	FG	CDEF	GH
2	FGH	FGH	FGH	FGH	DEF	FG
3	FGH	FGH	EFG	FGH	FGH	FGH
4	FGH	FGH	DEF	FGH	DEFG	H
6	FGH	H	FGH	FGH	BC	BCD
7	FGH	N/A	N/A	N/A	A	AB
8	FGH	N/A	N/A	N/A	AB	BCDE
9	FGH	N/A	N/A	N/A	FG	FG
10	FGH	N/A	N/A	N/A	FGH	FGH

Niraparib [μg/mL]	F7 [μg/mL]					
	0	7.5	10	12.5	15	17.5
0	GHIJ	GHIJ	GHIJ	GHIJ	GHIJ	GHIJ
1	GHIJ	GHIJ	EFGH	IJ	HIJ	CDEFG
2	GHIJ	GHI	GHI	HIJ	FGHI	BCDEF
3	GHIJ	FGH	HIJ	GHIJ	BC	BC
4	GHIJ	EFGH	HIJ	GHIJ	BCD	BC
6	GHIJ	HIJ	J	HIJ	AB	B
7	GHIJ	N/A	N/A	N/A	A	AB
8	GHIJ	N/A	N/A	N/A	AB	BCDE
9	GHIJ	N/A	N/A	N/A	DEFGH	EFGH
10	GHIJ	N/A	N/A	N/A	GHI	GHI

Niraparib [μg/mL]	F5-SM [μg/mL]				
	0	7.5	10	12.5	15
0	HI	HI	HI	HI	HI
4	HI	EFGHI	CDEF	A	GHI
6	HI	IJ	J	BCD	HIJ
7	HI	BC	AB	A	CDEFGH
8	HI	HI	CDEFG	CDE	FGHI
9	HI	HI	DEFGH	DEFGH	HI

Niraparib [μg/mL]	F7-SM [μg/mL]				
	0	7.5	10	12.5	15
0	G	G	G	G	G
4	G	ABCDEF	ABC	AB	ABCDE
6	G	ABC	ABCD	A	BCDEFG
7	G	ABC	AB	ABC	BCDEFG
8	G	CDEFG	ABCDE	CDEFG	CDEFG
9	G	FG	H	DEFG	EFG

Table S5. Different Upper case letters signify delta values that are significantly different from all combinations of pairs according to Tukey–Kramer honest significant difference (HSD; P ≤ 0.05). Delta values calculated according to Bliss model between the experimental (observed) and the calculated (expected) values of the synergistic interactions between F5 or F7 and niraparib on cell viability of HTB161 cells following combined treatments. Delta values are graphically presented in Figure 7.

		F5 [µg/mL]					
Niraparib [µg/mL]		0	15	17.5	20	22.5	25
	0	CDEF	CDEF	CDEF	CDEF	CDEF	CDEF
	4	CDEF	ABC	ABC	A	ABC	EFGHI
	8	CDEF	BCD	BCDEF	DEFG	CDEF	GHI
	12	CDEF	A	DEFGH	CDEF	BCDEF	GHI
	16	CDEF	BCDE	DEFG	FGHI	EFGHI	I
	20	CDEF	BCD	DEFG	EFGHI	DEFGH	HI
		F7 [µg/mL]					
Niraparib [µg/mL]		0	17.5	20	22.5	25	27.5
	0	CD	CDE	CDE	CDE	CD	CD
	10	CDE	ABC	CD	E	CD	CDE
	15	CD	CD	ABC	DE	BC	CDE
	20	CDE	ABC	CDE	CDE	ABC	C
	25	CD	AB	A	CD	C	BC
	27.5	CD	ABC	ABC	CD	CD	CD

Table S6. Different Upper case letters signify delta values that are significantly different from all combinations of pairs according to Tukey–Kramer honest significant difference (HSD; P ≤ 0.05). Delta values calculated according to Bliss model between the experimental (observed) and the calculated (expected) values of the synergistic interactions between F5, F7, F5-SM or F7-SM and gemcitabine on cell viability of HTB75 cells following combined treatments. Delta values are graphically presented in Supplementary Figure S3.

Gemcitabine [ng/mL]	F5 [μg/mL]					
	0	7.5	10	12.5	15	17.5
0	ABCDE	ABCDE	ABCDE	ABCDE	ABCDE	ABCDE
2.5	ABCDE	AB	ABCD	ABCD	EFGH	ABCDEF
5	ABCDE	ABCD	A	J	J	GHI
10	ABCDE	BCDEFG	DEFGH	IJ	CDEFGH	ABC
50	ABCDE	ABC	CDEFGH	FGHI	J	CDEFGH
100	ABCDE	AB	ABCDEF	HI	J	BCDEFGH

Gemcitabine [ng/mL]	F7 [μg/mL]					
	0	7.5	10	12.5	15	17.5
0	A	A	A	A	A	A
2.5	A	DEF	IJ	K	FGH	ABCD
5	A	BCD	HI	JK	ABCD	ABC
10	A	CDE	DEF	DEFG	AB	ABC
50	A	BCD	EFGH	IJK	GHI	ABCD
100	A	ABC	DEFG	HI	CDE	ABC

Gemcitabine [ng/mL]	F5-SM [μg/mL]					
	0	7.5	10	12.5	15	17.5
0	BC	BC	BC	BC	BC	BC
2.5	BC	AB	A	BC	BC	BC
5	BC	BC	C	BC	BC	BC
10	BC	ABC	ABC	BC	BC	BC
50	BC	AB	ABC	BC	BC	BC
100	BC	AB	AB	BC	BC	BC

Gemcitabine [ng/mL]	F7-SM [μg/mL]					
	0	7.5	10	12.5	15	17.5
0	CDEFGH	CDEFGH	CDEFGH	CDEFGH	CDEFGH	CDEFGH
2.5	CDEFGH	A	ABCD	EFGHI	BCDEFG	DEFGHI
5	CDEFGH	AB	DEFGHI	FGHIJ	BCDEFG	DEFGHI
10	CDEFGH	ABCDE	HIJK	DEFGHI	ABCDEF	DEFGHI
50	CDEFGH	ABC	IJK	JK	DEFGHI	DEFGHI
100	CDEFGH	A	EFGHI	K	GHIJ	DEFGHI
